# Methamphetamine-type stimulant use in Lao PDR: qualitative findings from users aged 15–25 years in Vientiane Capital and Vientiane Province

**DOI:** 10.1186/s12954-018-0222-1

**Published:** 2018-04-02

**Authors:** Vanphanom Sychareun, Bangone Santavasy, Niramonh Chanlivong, Andrea Fischer, Nicholas Thomson, Robert Power, Jo Durham

**Affiliations:** 1 University of Health Sciences, Vientiane, Lao People’s Democratic Republic; 2 Burnet Institute in Laos, Luangprabang Road, Building 06, 2A/03, Ban Sihom, Vientiane, Lao People’s Democratic Republic; 3 The Peter Doherty Institute for Infection and Immunity, University of Melbourne, Melbourne, Australia; 4 Research Institute of Health Sciences, Chiang Mai University, Chiang Mai, Thailand; 5 Johns Hopkins, Bloomberg School of Public Health, Baltimore, MD USA; 6 Faculty of Biomedical Sciences, School of Public Health, University of Queensland, Herston Road, Herston, Queensland 4006 Australia

**Keywords:** Methamphetamine, Yaba, Youth, Lao PDR, Harm reduction, Illicit drugs, STIs, Bourdieu

## Abstract

**Background:**

Methamphetamine is one of the most widely used illicit drugs in several Southeast Asian countries, including the Lao People’s Democratic Republic (PDR). The purpose of this study was to examine the socio-cultural context of its use in Lao PDR.

**Methods:**

We conducted a cross-sectional qualitative survey among young people, aged 15–24 years, who use methamphetamine (or Yaba as it is commonly known in the region) in Vientiane Capital and Vientiane Province, Lao PDR. In total, we conducted 21 in-depth interviews (6 female, 13 males and 2 transgender) and 8 focus group discussions with 47 participants (10 female, 29 male and 8 transgender). The data analysis drew on the theory and insights of the social theorist Pierre Bourdieu (1990, 1997) to understand the Yaba consumption practices among young Laotians.

**Results:**

Yaba consumption among young people in this study was used to enhance both productivity and pleasure. Its pleasure-producing affects were often linked to core Laotian notions of having fun, partying and being together. Its increased productivity effects on the other hand, were used to pursue goals related to self-betterment within an emerging market economy.

**Conclusions:**

This study highlights the complex interaction between agency and identity, structure, context and practices. In terms of public health policy and programme responses, developing proper interventions implies a good understanding of how young people interpret Yaba consumption, its dynamics and the relationships and resources that mediate the behaviours.

## Background

Amphetamine-type stimulants including methamphetamine as well as cannabis and opium, are among the most widely used illicit drugs used in the Southeast Asia region [1–4]. Within the region, methamphetamine is mainly available in tablet form or crystalline methamphetamine. Methamphetamine tablets, commonly known as ‘Yaba’ in the region, are small pills consisting of a mix of methamphetamine and caffeine. Crystalline methamphetamine, also called ‘crystal meth’, ‘ice’ or ‘shabu’, is usually of much higher purity than the tablet form. Methamphetamine is a central nervous system stimulant and can produce feelings of euphoria, perceived increases in confidence and physical strength and appetite suppression [5]. Methamphetamine use has also been found to increase libido, lower inhibitions, enhance sexual pleasure, prolong sexual intercourse [6, 7] and contribute to unsafe sexual intercourse [8]. Adverse effects of prolonged use can include anxiety, paranoia, psychosis, panic and cardiovascular issues including arrhythmias, intracranial bleeding and congestive heart failure [5]. The purpose of this paper is to examine Yaba use in young people living in Vientiane capital and Vientiane Province in the Lao People’s Democratic Republic (PDR), where its use is of increasing concern [9].

Situated in Southeast Asia, the Lao PDR, borders every country in the Greater Mekong Subregion (Thailand, Cambodia, Vietnam, China and Myanmar), with the northern part of the country forming part of the infamous drug-producing region known as the Golden Triangle [10]. Lao PDR has long been regarded as a major opium producer. In recent years, however, methamphetamine (or Yaba) in tablet form, has entered the market and is now estimated to be more widely used than opium. Initially introduced to East Asia during World War II to enhance soldiers’ performance, Yaba has been largely used by occupational workers such as bus and truck drivers. As countries in the region have experienced increased economic development, however, Yaba has expanded into youth culture [11], including in Lao PDR where young people's use of Yaba is of particular concern to the government [10, 12, 13]. For many young people, unlike opium, Yaba is linked with modern youth urban culture and associated with pleasure, improved physical activity and mental alertness [13]. Within the mainstream adult discourse, however, Yaba use is linked to the concerns about urban youth and a shift away from traditional values [13].

Yaba is thought to be the cheapest and most widely available illegal drug available in Lao PDR. Accurate data on use is scant with no available figures of those who are considered to be problem drug users (i.e. injecting drug use or long duration/regular use of opioids, cocaine and/or amphetamines [14]). A 2009 cross-sectional survey of Yaba users aged between 15 and 24 years in Vientiane Province (*n* = 443) reported the mean age of initiation was 16.4 years (SD = 2.475); inhalation or smoking (90%) were the main routes of administration with minimal rates of injecting [15]. Concomitant Yaba use with alcohol (98.6%, *n* = 436) and tobacco (86.7%, *n* = 384) was also reported [15]. Furthermore, at approximately 95% of people treated for drug use within the country in recent years, the proportion of people receiving treatment for methamphetamine tablets was the largest. Since 2013, the policy response has shifted from detention to community-based treatment, with approximately 98% of people treated for drug use at the Somsanga Treatment and Rehabilitation Center, in Vientiane capital, in 2013 and 2015 Yaba users. This is in contrast to the other treatment and rehabilitation centres in the country where most people are reported to be treated for heroin, alcohol, inhalants and cannabis use. Somsanga however was the only centre providing specialised treatment for Yaba and other amphetamine-type substances, and this may account for the difference. Since 2013 more treatment centres are now available that can treat people with Yaba addictions but the number of cases are not available [9].

While the pharmacological effects of Yaba are important in understanding young people’s use of the drug, socio-cultural context is also important in understanding how young Laotians experience its use. Yet, despite the increase in Yaba use, to date, there have been very few qualitative studies that have examined the socio-cultural context of Yaba consumption in the Lao PDR. In this paper, we present our qualitative findings from a larger study to illustrate the interplay between agency and identity, structure and young people’s Yaba use in Vientiane Capital and Vientiane Province, Lao PDR.

### Theoretical framework

To understand Yaba consumption among young people, the present study drew on the work of Pierre Bourdieu [16–18]. Three concepts that are fundamental to Bourdieu’s work are field, capital and habitus [17, 18]. A field for Bourdieu is a social space characterised by regularity and norms, in which actors struggle for reward, as in a game [17, 18]. It may be a specific profession or industry or a larger structure such as a social class grouping [19, 20]. Individuals live and act in overlapping fields, within which there are distinct social hierarchies and power relations, where certain agents have the power to set out the ‘rules of the game’ [17, 18, 20]. These fields develop historically and are both cultural and social phenomena with earlier structures, cultures and fields continuing to exist in some way as other fields evolve. Writing about the concept of field as a site of struggles, Moore [21] explains how dominant research disciplines have focussed on risk factors associated with drug use while ignoring the pleasure inducing affects of illicit drugs.

Capital is embodied in economic, cultural, social and symbolic forms and helps determine a person’s position in a social field [17, 18]. Economic capital refers to material and financial assets and other forms of income such as wages. Cultural capital describes the skills and knowledge that are considered legitimate and useful in the dominant society. Social capital relates to social connections, the utility of which depends on the depth and breadth of one’s social networks and the volume of material resources held by people within these networks [17, 18]. Social capital is not necessarily a given, but something that must be continually invested in, individually or collectively, consciously or unconsciously to establish or reproduce social relationships that have value [17]. A number of scholars have identified the role of social capital and social networks in producing and maintaining drug use [1, 22–25].

Symbolic capital is any form of capital that is given positive recognition by relevant actors within the field [17, 26] and can relate to any form of capital when it obtains an explicit or practical recognition in a specific field. These different capitals are inter-independent; effective deployment of one type can lead to the acquisition of additional or another type of capital. For Bourdieu, investment in these different capitals is a means to resources which are highly valued in capitalist societies. In particular, investment in social and cultural capital can be a means to increase economic capital.

The concept of habitus provides a means to understand social actions [21]. For Bourdieu, habitus is a personal set of cognitive, deeply engrained dispositions that guide what people think and how they act. Habitus develops through socialisation, with people who move through similar social contexts acquiring similar habitus, allowing the development of a ‘logic of practice’, inculcating in people a ‘worldview’, based upon their position, helping to explain how we act in the world, what we aspire to and our consumption preferences [18]. In other words, habitus includes propensities to act and are acquired over the life course, with action learned in a specific social environment. Habitus provides a way of understanding localised strategic actions and is the link between individual agency, capital and the structural constraints that shape health-seeking practices [22]. Lunnay and colleagues in a study of alcohol consumption among young females, showed how social drinking was part of and reflected participants’ ‘habitus’ and socially constructed view of the world [27]. They explain how young female drinking provides an opportunity for gaining symbolic capital conferring upon the young female a sign of distinction within a wider habitus [27].

Drawing on the work of Bourdieu, Rehbein argues that society can be understood as a bundle of layers that comprise social structures from different historical times. Rehbein [28] calls these layers ‘sociocultures’ within which different recourses are ascribed value. These sociocultures also contribute to the development of habitus and given that each socioculture is socially differentiated, the habitus acquired within one socioculture is also differentiated.

## Methods

The study is part of a larger research project which included a survey and qualitative interviews. This paper documents the qualitative data collected through in-depth interviews and focus groups discussions (FGDs).

### Setting

The study was undertaken in Vientiane Capital and Vientiane Province. Vientiane Capital is the largest city in the Lao PDR and the centre of its economic growth. An increasing number of young people are migrating from rural areas to Vientiane Capital and Vientiane Province for work or study [23]. Six districts in Vientiane Capital (Chanthabuly, Sikhottabong, Sisattanak, Saysetha, Saythany and Hadxayphong) and five districts in Vientiane Province (Phonghong, Viengkham, Thourakhom, Keooudom and Vangvieng) were selected for this study based on high estimates of a current number of Yaba users in these areas. In each site, ‘hot spots’ where young people and drug users congregate were identified and mapped.

### Sampling

This study was part of a larger study, which included a survey and qualitative interviews. After identifying and mapping places with five peer educators, where young Yaba users gather, purposive, snowball sampling was used to recruit participants. The inclusion criteria for study participants were (1) 15–24 years of age, (2) Lao citizens living in the province for at least 3 months, (3) current or former Yaba user (current users were defined as those who used Yaba at least once during the 3 months prior to the survey (we selected 3 months because we wanted to capture as much as possible the events of Yaba use); former users were defined as those who used Yaba at least once in the 2 years prior to the survey) and (4) ability to communicate with the research team verbally in Lao. For the qualitative component, which is the focus of this paper, participants were recruited from the quantitative component of the survey (*n* = 443) to reflect the gender make-up of the quantitative component. Reflecting the initial survey, we recruited a mix of students and those who had already graduated and a mix of genders.

### Data collection

In-depth face-to-face interviews and FGDs were conducted to gain a better understanding of the socio-cultural context of Yaba use. Using an interview guide, aspects such as initiation into Yaba consumption, consumption patterns, reasons for use, and sexual pleasure and sexual risk under the influence of Yaba were explored. We included sexual risk because unprotected sex and multiple sexual partners have been associated with drug use. The interview guides used for the in-depth interviews and FGDs were open-ended to allow participants to focus on the issues that were important to them and to give the interviewer the flexibility to follow up or clarify on certain areas of interest [29, 30].

A total of eight FGDs were undertaken (one FGD for transgender, two FGD for females, two mixed group of male and females, two mixed group of male, female and transgender and two FGDs for males). Individual interviews were conducted with 20 Yaba users (6 females, 12 males and 2 transgender people) selected by the peer educators.

Interviews and FGDs were conducted in places convenient for the participants, such as temples, referral centres and the peer researchers’ homes. Interviews and FGDs were audio-recorded using a digital recorder, transcribed by a professional transcriptionist and translated into English. Personal identifiers were removed. Discussions ranged from 45 min to 3 h. Participation was voluntary, and written consent was obtained in every case. Reimbursements (condoms and 50,000 Kip in cash, equivalent to USD 6.5) were provided to study participants to compensate for their time.

### Data analysis

The transcripts were read and re-read and manually coded using a combination of inductive and deductive approaches [29, 31]. Key issues, concepts and themes were identified a priori including, for example, the role of peers and social networks, this did not prevent, however, other themes emerging as we read and re-read the transcripts focusing on the meaning of the participants and specific observations to detect themes and patterns in the data [32]. We subsequently used a deductive approach drawing on Bourdieu’s theory of habitus, field and capital, focusing for example on resources, disposition, choices and the context of Yaba use to help explain the findings [26]. The objective of the analysis was to use Bourdieu’s concepts to reveal participants’ specific interests, identify how individuals were positioned within their social field and their access to capital (e.g. social, economic, cultural), the strategies they employed in the accumulation of capital and how these interests reflected or become sources of capital [26]. Throughout the analytic process, researchers moved back and forth between the entire data set and the coded extracts discussing relevant issues as they arose with analysis continuing throughout the writing process [33].

### Ethics

Ethical reviews were conducted by the steering committee established to oversee this study and the Ministry of Health/Ministry of Foreign Affairs. The research proposal received formal ethical approval from the National Ethical Committee for Health Research, Ministry of Health in March 2008 (Ref: 168/NECHR), Lao PDR. Transcripts were de-identified to ensure that the study participants could not be traced.

## Results

### Demographics

Twenty people (6 female, 12 male and 2 transgender) completed the in-depth interviews. Forty-seven people (10 female, 29 male and 8 transgender) participated in a total of eight FGDs. Of those interviewed, 60.8% were male, 24.6% were female and 14.6% were transgender, all aged between 15 and 25 years. Just over half (53.6%) were from Vientiane Province, and the remaining 46.4% were from Vientiane Capital. Twelve participants were students, and the rest were either employed in the informal sector including small trade, manual labour and farming or unemployed. One person referred to her occupation as being a housewife. The majority of participants had completed secondary level education.

Most participants started using Yaba around the age of 15 years old, with a range from 12 to 22 years old. Interviewees had used Yaba for 5 years on average (ranging from a few months to 14 years). Among the Yaba users surveyed, 45.8% admitted to being current users, with the average duration between first use and becoming a regular user (defined as use at least once a month) of 58.7 days. Most reported using used Yaba approximately 2–3 days per week in the 3 months preceding the study, typically with friends, at a partner’s house or in their own homes, mainly for recreation and to increase productivity. None of the respondents reported injecting Yaba.

### Yaba use, social networks and pleasure

Many of the respondents expressed a strong sense of being an active part of a social group, and almost all were introduced to Yaba by their friends, with the desire for group inclusion an instigator for Yaba use. While the participants operated in different fields, the sense of belonging, being supported, accepted and having fun were important in being part of a social group that used Yaba, and where there was an inherent acceptance of its use. Participants described buying or being given Yaba from friends with no participants reporting buying Yaba from strangers.

In Bourdieu’s terms, Yaba use became naturalised due to the particular social influences and interactions within participants’ networks. This link between social structure and practice shows how cognitive and motivating structures related to Yaba use became internalised as a viable option for being included in and having fun within a social group. The following interview excerpt helps illustrate comments made by both males and females. It helps to highlight the social pressure within participants’ peer groups or fear of being excluded and the potential of losing social capital by not acting according to the ‘rules of the game’ if they did not take Yaba.

‘I use Yaba because my friends persuaded me to. If I don’t use it my friends will not want to meet with me or socialise with me, or they will not call me to go out with them anymore.’ (Male, 21 years old)

Most of the respondents in this study smoked Yaba. With this method, the pills were usually heated with a cigarette lighter in a piece of foil shaped as a boat and the fumes inhaled. This usually took place with friends in social or recreational settings such as a friend’s house, a school toilet or dormitory, a nightclub or in a rice field. While Yaba may be ingested orally, smoking was preferred not only because of the greater euphoric effect but also because smoking Yaba with friends formed a ritual or social activity that added to a sense of solidarity. In this way, Yaba use was linked to being with friends and enjoyment, suggesting a desire for social capital one motivator for its use. Within Lao PDR, across different milieu being with friends and enjoying oneself, having fun (muan), partying (bun), being together and relaxing are fundamental to one’s habitus and basic understanding of a good life.

### Yaba use and symbolic capital and gender identities

Symbolic capital relates to anything that bolsters social status and is recognised by social agents as having value in a social context. Not belonging to the political or economic elite, the relatively low cost of Yaba also allowed participants to purchase a commodity initially used by young, well-connected young people, elevating their status, allowing them to consume a ‘modern’ drug (unlike opium for example), conferring symbolic capital within the young person’s social network. In Bourdieusian terms, by engaging in Yaba consumption, participants were entering into a game-like scenario where they could make social gains through Yaba consumption.

Some participants talked about how Yaba use gave them other attributes which they valued, such as becoming more extroverted and sociable and able to ‘dance all night’ (male respondent, 21 years). They talked about being more productive and about having better academic performance—attributes valued in the Laotian consumer society—and having increased self-esteem and confidence. Within this context, Yaba use could be transformed into a value-laden commodity that as with ‘dancing all night’ conferred social distinction or reputation, through a way of being that represented the ‘popular aesthetic’ within participants social networks [16] as well as having fun.

### Yaba use, productivity and gender identities

Yaba consumption enabled participants to balance the demands of ‘modern’ and ‘traditional’ society with its performance-related effects used to maximise individual achievement at school and work. Participants felt Yaba use helped them manage the worries associated with earning a living and participating in a consumerist culture, while at the same time conforming to traditional norms of masculinity and femininity. While both males and females talked about Yaba use creating increased sexual desire and pleasure, males also emphasised protracted sexual encounters boasting about their virility and sexual prowess through sexual performance, and a masculine habitus, with Yaba also a means to new and pleasurable experiences.

‘Yaba has an effect on sexual desire because it helps prolong sexual intercourse. One can have sex all night.’ (Male, aged 20 years)

Some female respondents mentioned they used Yaba because they valued its weight-reducing properties. This may be related to the growing pressure on young Lao women to be slim in the modern Lao society.

‘I use Yaba with my friends for more energy and losing weight.’ (Female respondent, 19 years.)

While the wider social and family culture did not sanction Yaba use, its stimulating effects were reported to enable users to perform better in most of their daily activities including taken for granted gendered practices. In balancing the demands of the market economy and maintaining their gendered roles, some female respondents observed for example, that Yaba use gave them the energy to complete their traditional household tasks, with a practice rooted in a gendered habitus. As one person explained:

‘After taking Yaba, I can work for longer and am not lazy, so when my parents ask me to do housework I can do everything.’ (Female respondent, 20 years.)

In this way, they created a positive self- and family-identity by conforming to gender norms. These examples show how Yaba use can contribute to a positive feedback loop, tipping young people’s attitudes and dispositions towards the positive aspects of Yaba use, despite its unacceptability among family and the broader community. Many respondents noted, for example, that even though Yaba consumption was an important part of belonging to a peer group, its use meant they were often alienated in other social spheres where Yaba use was derided. Thus, while Yaba use built social capital in particular fields and related to fun, pleasure, belonging and increased productivity, it could also be a negative experience related to social dissociation and marginalisation, due to its inconsistency with cultural ideals and the habitus of other social groups. As noted by one:

‘We are not like other members of the community, we are excluded and they do not pay any attention to us.’ (Male respondent, 25 years)

As seen earlier, however, Yaba use was not necessarily taken as a way to deviate from social norms. Rather, Yaba use was mainly based on the desire to acquire valued forms of capital, avoid being excluded by one’s social network and perform in relation to both gendered habitus and the demands of the market economy.

In spite of the potential risk associated with Yaba use, participants said that these risks had not been considered when they first tried Yaba. Participants also reported that Yaba consumption was seen as a legitimate way of reducing other health risks such as mental stress due to school or family pressures, tiredness, interpersonal conflict and poor relationships with parents. The following excerpt provides an example of this:

‘I had an argument with my mother on my birthday, then I went to see my friends and they asked what happened. Then they persuaded me to use Yaba …… I felt relaxed and stopped worrying about my family problems.’ (Male respondent, 15 years.)

Alcohol, tobacco and marijuana were mentioned as being consumed in conjunction with Yaba. Somewhat paradoxically, given the relatively high level of acceptance of alcohol use within Lao PDR, some young people said they took Yaba to reduce the risks of being drunk when taking alcohol. For these youngsters, Yaba use was considered valid and desirable within their social context, while being drunk was not. This shows how Yaba use allowed participants to distinguish themselves from young people in other scenes, while earning status within their own. In this way, Yaba users in this study enacted particular logic of practice whereby Yaba use was acceptable but excessive alcohol use was not.

The effects of Yaba consumption on sexual risk were different in men and women, although both reported multiple sexual partners and inconsistent condom use when engaging in sexual intercourse under the influence of Yaba. For females, in particular, negotiating condom use is often difficult, regardless of Yaba or other substance use. This relates to the gendered rules of the game or structure of the field of heterosexual encounters that typically precludes assertive sexual demands such as condom use. Bourdieu (1990) called this ‘amor fati’ or ‘love of one’s fate’, whereby social agents refuse to do something that is already denied to them (in this case successfully negotiate condom use) and is reflective of cultural rules entangled within a given field of social interaction with female bodies embedded in socio-cultural norms in a patriarchal culture. The concept of habitus also helps to explain how having multiple sexual partners and unprotected sex can become second nature.

Accessing economic capital was often necessary for securing access to Yaba and while relatively cheap, a number of young men and a few women in this study reported either exchanging sex for Yaba or exchanging sex for money to buy Yaba. The interviews suggested however that this transactional sex was informal and occasional, occurring within participant’s social networks.

Some respondents had considered quitting Yaba. Family or community disapproval was the main reasons for considering this. Participants who said they had tried to stop before had tried to do so by staying away from friends, avoiding areas where they knew there would be dealers and engaging in other recreational activities or meditation. While family understanding and support were seen as a crucial element of giving up Yaba, many users were estranged from their families or were in frequent conflict with them because of their Yaba use. Efforts to abandon Yaba were therefore often thwarted as seeking social inclusion and a sense of belonging and valuing the productive effects of Yaba they returned to their peer networks and resumed Yaba use.

## Discussion

There are few qualitative studies examining Yaba consumption in young people in the Lao PDR, an issue that is rapidly becoming a public health concern [2]. Our findings support those of others [1, 25, 34], which have similarly highlighted the inextricable link between social networks and drug use, and the association between Yaba and lowered inhibitions, social bonding, fun and enjoyment and can confer distinction to groups and strengthen ties to friends [11, 21]. Of concern, is most of the participants first used Yaba when they were 15. The place of Yaba use was similar across genders and included schools, workplaces and entertainment venues. Yaba’s performance-enhancing effects were directed towards improving performance through academic achievement (cultural capital), increasing economic activity and pleasing family by undertaking gendered practices. Through these processes, Yaba users further developed a taste for Yaba because of its use-value. These findings on the relationship between drug use pleasure and performance echo those found elsewhere in the region [13, 35–39] and more globally [21, 27].

In many of our interviews, Yaba use was linked with the core Laotian concepts of pleasure, including having fun, partying, being together and relaxing. Rehbein suggests that these four concepts can be summarised under the term ‘pen kan eng’, which approximately translates to letting go or acting according to one’s habitus [28]. The pleasure-producing effects of Yaba therefore, can be seen to link Yaba users to the socioculture in which their habitus has developed. At the same time, it can be linked to the emerging consumer milieu whereby Yaba is a pleasure commodity and part of youth urban culture. Unlike their parents, young Lao living in urban areas are growing up in an increasingly consumerist culture with increased competitiveness [13, 40]. The extra physical energy provided by Yaba helped users work hard as they learn to navigate this new environment and its exhortations to economic competitiveness [13, 28, 40, 41].

Drug use and the role of social networks observed in this study have been well documented [1, 22, 24, 25, 34, 42], and as in other studies, the internalisation of peer norms was evident in participants’ accounts. In particular, initiation of Yaba use revealed participants’ desire for the maintenance of social capital, with many expressing initially consuming Yaba to avoid social isolation. Bourdieu’s work [16–18] helps us to understand how this happens. Individuals, alone or collectively, consciously, or unconsciously, invest in developing networks of relationships that can be used in the short or longer term [17]. Within these networks, young people’s beliefs, in this case about Yaba consumption, become part of their social relationships, conferring status and inclusion within a specific social and cultural context and the normalisation of Yaba use [43]. Rather than making practices acceptable to the wider society, or pursing social capital for economic gain as conceptualised by Bourdieu [17, 18], however, Yaba users converted economic capital (buying Yaba) into social capital or used their social capital (or a combination of economic and social capital to access Yaba) within a particular subgroup solidifying the ties to their Yaba-taking friends, and defining separation from those who do not. A similar pattern has been observed in adolescent tobacco smoking [44]. This type of capital is similar to Thornton’s [45] term ‘subcultural capital’, whereby clusters of young people organise around specific activities, mindsets, tastes and styles that define their distinctiveness and are highly sought after by the members of a subculture but may be criticised by members of other cultural groups.

The concept of peer pressure has been a prominent concept in drug research with the debate around whether social networks are important because of social selection or social influence on drug use [22, 46, 47]. In the present study, when discussing initiation to Yaba, participants typically said they were ‘persuaded’ by friends and felt that not taking Yaba would result in group exclusion, suggesting strong peer pressure. In this sense, Yaba as a symbolic boundary of group inclusion is formed through the routine practices of members with non-users on the ‘outside’ [45]. This is in contrast to a study of participants aged 18 to 61 years in Florida where marijuana use was an important part of friendship identity, but, unlike the present study, quitting marijuana did not lead to group exclusion [24]. In a review of the evidence, Coggans and McKellar concluded that peer preference rather than peer pressure is a more accurate presentation of the role of a peer in drug use initiation [46]. Lunnay and colleagues [27] applying the work of Bourdieu to female alcohol consumption, reported that participants consistently rejected the idea of peer pressure. The suggestion of peer influence in our study may reflect different cultural patterns and habitus compared to where other studies have been performed. The young age of our participants at the time of initiation may also mean they felt more peer pressure [22]. Reinforcing factors such as ‘having fun’ and being more confident may also play a role, although negative attitudes from wider community members and family did not necessarily stop Yaba use. As we have elucidated drawing on the theory of Pierre Bourdieu, however, practices are a result of individual agency, mediated by the cultural and social structures that determine practice and the socio-cultural meanings they ascribe to objects [48]. Given young people’s social groups or milieu are likely to be congruent, peer ‘persuasion’ may be more accurately interpreted as peer preference and an area for further research.

There are some potential limitations to this study. Considering that Yaba use was self-reported and was not confirmed with other markers of consumption, this may have created some reporting biases. Also, although the use of peer researchers is a strength of the study that facilitated access to an otherwise difficult-to-reach population, there is the possibility that the use of such peers introduced subjective bias into the study. It is also important to note that this study was conducted mainly in urban areas where Yaba are used for recreation and to facilitate work and study. This might differ from the patterns of use in rural areas where it is likely to be primarily used to enhance the productivity of workers in the fields. In addition, with our sample size, it was not possible to develop a more finely grained analysis of specific subgroups of users, but we were able to identify underlying patterns. Finally, transcripts were translated into English, and it is possible that the meaning could have been lost or distorted in the process.

## Conclusions

The paper provides insights about the context and patterns of Yaba use among young people in Lao PDR. It shows the positive symbolic value that participants allocate to Yaba. This is in stark contrast to the negative symbolism assigned by their families and the community. In terms of public health policy and programme responses, the work of Bourdieu helps to highlight that the understanding of young people’s practices, such as illicit drug use, needs to take into consideration that they are agents of their own lives, pursuing their own trajectories, situated within their own socio-economic, cultural and relational worlds. What this implies is that developing interventions to minimise Yaba use should examine in-depth how young people interpret and understand drug use, its dynamics and the relationships and resources that mediate practices. From this perspective, the demonising of Yaba users is unlikely to be effective. Ultimately, what is needed is a more nuanced, multi-faceted understanding of the reasons behind young people’s consumption choices in order to design more effective public health strategies. Such strategies will need to take account of the social, cultural and economic drivers of unhealthy consumption practices.

## Abbreviations

ATS: Amphetamine-type stimulants
AusAID: Australian Agency for International Development
FGD: Focus group discussions
PDR: People’s Democratic Republic
